# Comparing modalities for risk assessment in patients with pulmonary lesions and nondiagnostic bronchoscopy for suspected lung cancer

**DOI:** 10.1186/s12890-022-02181-x

**Published:** 2022-11-24

**Authors:** Diana H. Yu, Majid Shafiq, Hitesh Batra, Marla Johnson, Bailey Griscom, Janna Chamberlin, Lori R. Lofaro, Jing Huang, William A. Bulman, Giulia C. Kennedy, Lonny B. Yarmus, Hans J. Lee, David Feller-Kopman

**Affiliations:** 1 Department of Medicine, Division of Pulmonary, Critical Care, Allergy, and Sleep Medicine, University of California, San Francisco, San Francisco, USA CA; 2 Brigham and Women’s Hospital, Department of Medicine, Division of Pulmonary and Critical Care Medicine, Boston, MA USA; 3 Department of Medicine, Division of Pulmonary and Critical Care Medicine Birmingham, University of Alabama at Birmingham, Birmingham, AL USA; 4 Veracyte, Inc., South San Francisco, CA USA; 5 Division of Pulmonary and Critical Care Medicine, Section of Interventional Pulmonology, Johns Hopkins University School of Medicine, Baltimore, MD USA; 6 Department of Medicine, Division of Pulmonary and Critical Care Medicine, Dartmouth College, Hanover, NH USA; 7 UCSF Medical Center, 505 Parnassus Ave, 9414 San Francisco, CA USA

**Keywords:** Lung cancer, Risk assessment, Bronchoscopy

## Abstract

**Background:**

Bronchoscopy is commonly utilized for non-surgical sampling of indeterminant pulmonary lesions, but nondiagnostic procedures are common. Accurate assessment of the risk of malignancy is essential for decision making in these patients, yet we lack tools that perform well across this heterogeneous group of patients. We sought to evaluate the accuracy of three previously validated risk models and physician-assessed risk (PAR) in patients with a newly identified lung lesion undergoing bronchoscopy for suspected lung cancer where the result is nondiagnostic.

**Methods:**

We performed an analysis of prospective data collected for the Percepta Bronchial Genomic Classifier Multicenter Registry. PAR and three previously validated risk models (Mayo Clinic, Veteran’s Affairs, and Brock) were used to determine the probability of lung cancer (low, intermediate, or high) in 375 patients with pulmonary lesions who underwent bronchoscopy for possible lung cancer with nondiagnostic pathology. Results were compared to the actual adjudicated prevalence of malignancy in each pre-test risk group, determined with a minimum of 12 months follow up after bronchoscopy.

**Results:**

PAR and the risk models performed poorly overall in the assessment of risk in this patient population. PAR most closely matched the observed prevalence of malignancy in patients at 12 months after bronchoscopy, but all modalities had a low area under the curve, and in all clinical models more than half of all the lesions labeled as high risk were truly or likely benign. The studied risk model calculators overestimate the risk of malignancy compared to PAR, particularly in the subset in older patients, irregularly bordered nodules, and masses > 3 cm. Overall, the risk models perform only slightly better when confined to lung nodules < 3 cm in this population.

**Conclusion:**

The currently available tools for the assessment of risk of malignancy perform suboptimally in patients with nondiagnostic findings following a bronchoscopic evaluation for lung cancer. More accurate and objective tools for risk assessment are needed.

**Trial registration:**

not applicable.

## Rationale

In a patient with a newly identified lung lesion, an accurate assessment of the likelihood of lung cancer is needed to inform subsequent management decisions. Most small nodules, whether detected incidentally or through lung cancer screening, will ultimately prove to be benign[1, 2]. Larger lesions (> 30 mm) are more likely to be cancer, but benign lesions in this size range are not uncommon. To minimize harm and maximize benefit, there must be effort to avoid unnecessary invasive procedures in patients with benign disease while promptly identifying and treating those with early-stage lung cancer[3]. Current guidelines recommend that physicians evaluate a pretest probability of malignancy as an initial step in decision-making[4]. This estimate is used to determine the appropriate management plan: surveillance in very low risk patients, surgical resection in high-risk patients for those with early-stage disease, and non-surgical tissue sampling for those of intermediate risk of malignancy.

There is considerable heterogeneity in the population of patients with indeterminant lung lesions, and the current methods for risk assessment have limitations. Guidelines recommend use of either a risk model or physician-assessed risk (PAR)[4, 5]. Risk models estimate the probability of malignancy utilizing clinical and radiographic features, but their accuracy is dependent on the degree to which an individual patient reflects the cohort in which the model was developed and the prevalence of malignancy in that cohort[6]. An individual patient can have widely discordant risk estimates when assessed using multiple models. Moreover, the risk estimates of all these models are largely driven by size[7]. Very small cancers are more likely to be classified as low risk, whereas larger benign lesions are more likely to be classified as high risk. PAR has its own limitations and will be highly dependent on level of experience[6, 8].

Pulmonologists frequently elect to proceed with non-surgical tissue sampling in patients across the spectrum of risk[5, 9], often because of weighing additional factors such as patient preference. For airway accessible lesions, bronchoscopy is generally the preferred sampling modality[10] due to its lower risk of complications[4], but one must contend with the fact that bronchoscopy is frequently nondiagnostic, even with modern navigational platforms and robotic approaches[4, 10–12]. An accurate assessment of the risk of malignancy is critical to determine how best to proceed with these patients.

In this study, we assess the accuracy of three validated pulmonary nodule calculators and PAR in a cohort of patients who had a non-diagnostic bronchoscopy for a new pulmonary lesion.

## Methods

### Data source and study design

We performed a retrospective analysis of the baseline prospective data collected for the Percepta Bronchial Genomic Classifier Multicenter Registry[13]. Percepta Bronchial Genomic Classifier (BGC) is a molecular test that uses genomic information from normal-appearing epithelial cells from right mainstem bronchial brushings to further risk stratify the likelihood of malignancy in a lung lesion in the event of a nondiagnostic bronchoscopy. The classifier incorporates pre-bronchoscopy assessment of the risk of malignancy to provide accurate post-test risk and has been validated in lung nodules (≤ 30 mm) and lung masses (> 30 mm). The multicenter registry involved prospective collection of data at 35 U.S. centers and was designed to observe physician management of pulmonary lesions following a nondiagnostic bronchoscopy to gauge the impact of the classifier on decision-making. All patients underwent independent adjudication to determine, where possible, a final diagnosis (benign vs. malignant). This study represents a retrospective analysis of the accuracy of PAR and three previously validated nodule models, Mayo Clinic, Veteran’s Affairs (VA) and Brock, with respect to the final diagnosis in this cohort. Given that the registry was originally designed to collect comprehensive data only on those patients who received a classifier result because of a nondiagnostic procedure, our study cohort was restricted to that subset of patients.

### Patient characteristics

All patients enrolled in the registry study underwent flexible bronchoscopy for evaluation of an indeterminant lung lesion at one of 35 centers in the U.S. Lesions were identified during an evaluation of symptoms, were found incidentally, or were identified on low-dose CT screening for lung cancer. All patients were former or current smokers. Patients with a prior or concurrent history of lung cancer were excluded. Patients without complete information needed for adjudication of the three risk models (age, gender, smoking status, years since quitting, family history of lung cancer, presence of emphysema) were also excluded. Patients were deemed not eligible for adjudication if they had less than 12 months follow up, the classifier testing was outside of indication, or for a lack of a valid informed consent for the registry study at enrollment.

### CT **imaging**

All CT images underwent central panel review (DY, MS, HB) to provide standardized nodule characteristics and measurements to minimize site to site variability in radiographic interpretation. Reviewers were blinded to the final diagnosis. Lesions were characterized with respect to size, location (central/peripheral; upper/lower lobes), presence or absence of spiculation, nodule density (ground glass, partially solid, or solid), and nodule count. Patients without available CT imaging for analysis were excluded from the study.

### Adjudication of diagnoses (Benign versus malignant nodule)

Diagnosis of a benign or malignant nodule was determined through an adjudication process that had previously been performed on the entire Percepta Registry Cohort. An expert, three-member panel of pulmonologists (HJL, LBY and DFK) was convened to arbitrate a benign, malignant, or inconclusive consensus diagnosis. Panel members were provided with de-identified patient information with at least 12 months follow-up, and the panel members were blinded to the Percepta BGC results. A benign diagnosis was assigned in cases with (1) resolution or reduction in size of the nodule on surveillance imaging; (2) a definitive alternative benign diagnosis; or (3) nodule stability for ≥ 12 months and determination by the panel that the patient had no further suspicion of malignancy. This study relied upon one-year stability of the nodule based upon prior studies that have found one-year nodule stability to be predictive of stability at two years[12, 14]. A malignant diagnosis was assigned in cases with pathology reports confirming malignancy, or in cases where there was a documented plan to treat a patient with stereotactic body radiation therapy (SBRT) for presumed lung cancer without tissue confirmation. In cases where the adjudication panel could not determine a definitive malignant or benign diagnosis due to insufficient information (typically missing CT results at 12 months), patients were assigned a label of nondiagnostic.

### Physician risk assessment

The physician risk assessment used in the current study was that of the pulmonologist who performed the original bronchoscopy. Physicians were not given any specific guidance as to how to determine risk of malignancy, although use of a risk model calculator to inform PAR was permitted and documented. Only 13% of physicians in the registry study chose to use a risk calculator[13]. All data was collected prior to the procedure.

### Risk models

Centrally reviewed nodule characteristics and clinical history provided by the enrolling physicians were used as inputs to generate the estimated risk of malignancy using three risk models: Mayo Clinic, Veterans Affairs (VA) and Brock[4, 15–17]. Of the four Brock models, Brock 2b was chosen for this study because it had the best performance in our data set (data not shown). The three models chosen for this analysis were selected because they are considered to be well-validated in external cohorts[6]. Each uses linear regression to establish a point estimate for risk of malignancy. Results from each model were labeled according to the three risk categories used in the Registry Study: low risk (probability of malignancy < 10%), intermediate risk (probability of malignancy 10–60%) or high risk (probability of malignancy > 60%).

### Statistical analysis

The accuracy of the risk categorization for each model and for PAR was assessed by analyzing the actual prevalence of cancer among the patients placed into that category. Where the observed prevalence did not fall within the range specified for each risk group, a one-tailed *p-*test was used to determine if observed values were significantly different from the bound of that interval. A receiver operating characteristic (ROC) curve was generated for each model and for PAR, with calculations of area under the curve (AUC) for each modality. The data was analyzed combined and separately for lung nodules (lesions ≤ 30 mm) and lung masses (lesions > 30 mm).

## Results

We identified 1273 patients in the Percepta Registry Study as having newly identified lung lesions on CT who were referred for bronchoscopy, 1245 of whom underwent the procedure. In the group who underwent bronchoscopic biopsy, 496 patients (40%) were diagnosed with malignancy and 749 (60%) had a nondiagnostic procedure. Among the patients with a nondiagnostic result, 349 (47%) were deemed ineligible for adjudication. The reasons included less than 12 months follow up (31%); out of indication, most often due to an inadequate smoking history (20%); lack of valid patient consent for study participation (17%); no result on Percepta BGC testing (13%); missing clinical data (12%); patient death at < 12 months unrelated to lung cancer (4%); and lost to follow up (3%). The remaining 400 patients (53%) were adjudicated. An additional 25 patients were excluded because an initial CT was not available for review.

The characteristics of the 375 patients in the study cohort are presented in Table 1. The cohort included 125 patients (33%) with a final diagnosis of malignancy, 152 (41%) patients with a benign diagnosis, and 98 patients (26%) with no definitive diagnosis.

**Table 1:** Clinical and radiographic characteristics of the 375 patients in the study cohort

	Benign, N = 152^1^	Malignant, N = 125^1^	Unknown, N = 98^1^	p-value^2^
**Age**	62 (55, 69)	68 (63, 75)	65 (57, 71)	< 0.001
**Sex**				0.006
Female	60 (39%)	73 (58%)	50 (51%)	
Male	92 (61%)	52 (42%)	48 (49%)	
**Physician Assessed Risk**				< 0.001
High	27 (18%)	54 (43%)	9 (9.2%)	
Intermediate	107 (70%)	65 (52%)	71 (72%)	
Low	18 (12%)	6 (4.8%)	18 (18%)	
**Smoking Status**				0.4
Current	57 (38%)	45 (36%)	45 (46%)	
Former	94 (62%)	80 (64%)	53 (54%)	
Never	1 (0.7%)	0 (0%)	0 (0%)	
**Pack Years Smoked**	34 (17, 50)	40 (23, 54)	35 (20, 50)	0.10
**Years Since Quit**	6 (1, 22)	6 (2, 18)	1 (0, 14)	0.2
**Nodule Length (mm)**				0.001
(0–10]	18 (12%)	9 (7.2%)	15 (15%)	
(10–20]	53 (35%)	63 (50%)	43 (44%)	
(20–30]	29 (19%)	33 (26%)	12 (12%)	
(30+]	52 (34%)	20 (16%)	28 (29%)	
**Nodule Location**				0.2
Central	13 (8.6%)	4 (3.2%)	8 (8.2%)	
Peripheral	139 (91%)	121 (97%)	90 (92%)	
**Lobe Location**				0.7
Lower	56 (37%)	45 (36%)	33 (34%)	
Middle	16 (11%)	8 (6.4%)	8 (8.2%)	
Upper	80 (53%)	72 (58%)	57 (58%)	
**Nodule Density**				0.5
Ground glass opacity	8 (5.3%)	4 (3.2%)	7 (7.1%)	
Partial solid	9 (5.9%)	13 (10%)	7 (7.1%)	
Solid	135 (89%)	108 (86%)	84 (86%)	
**Nodule Margins**				< 0.001
Irregular	95 (62%)	58 (46%)	56 (57%)	
Lobulated	6 (3.9%)	5 (4.0%)	5 (5.1%)	
Smooth	15 (9.9%)	3 (2.4%)	12 (12%)	
Spiculated	36 (24%)	59 (47%)	25 (26%)	

Patients with a malignancy diagnosis were older than those with a benign diagnosis (mean age 68 vs. 62 years) or without a diagnosis (mean age 65 years) (p < 0.01) and were more likely to be female (p < 0.01). The groups differed by nodule size, with more patients ultimately diagnosed with malignancy having nodules within the range of 1-3 cm compared to less than 1 cm (76% for malignant vs. 54% for benign and 56% for nondiagnostic; p < 0.01). Patients with cancer were more likely to have spiculated lesions (47% for malignant vs. 24% for benign and 26% for nondiagnostic; p < 0.01). Most notably, patients with cancer were less likely to have large (> 3 cm) lesions.

### Cancer Prevalence in Low, Intermediate and High Risk categories

**Fig. 1:**
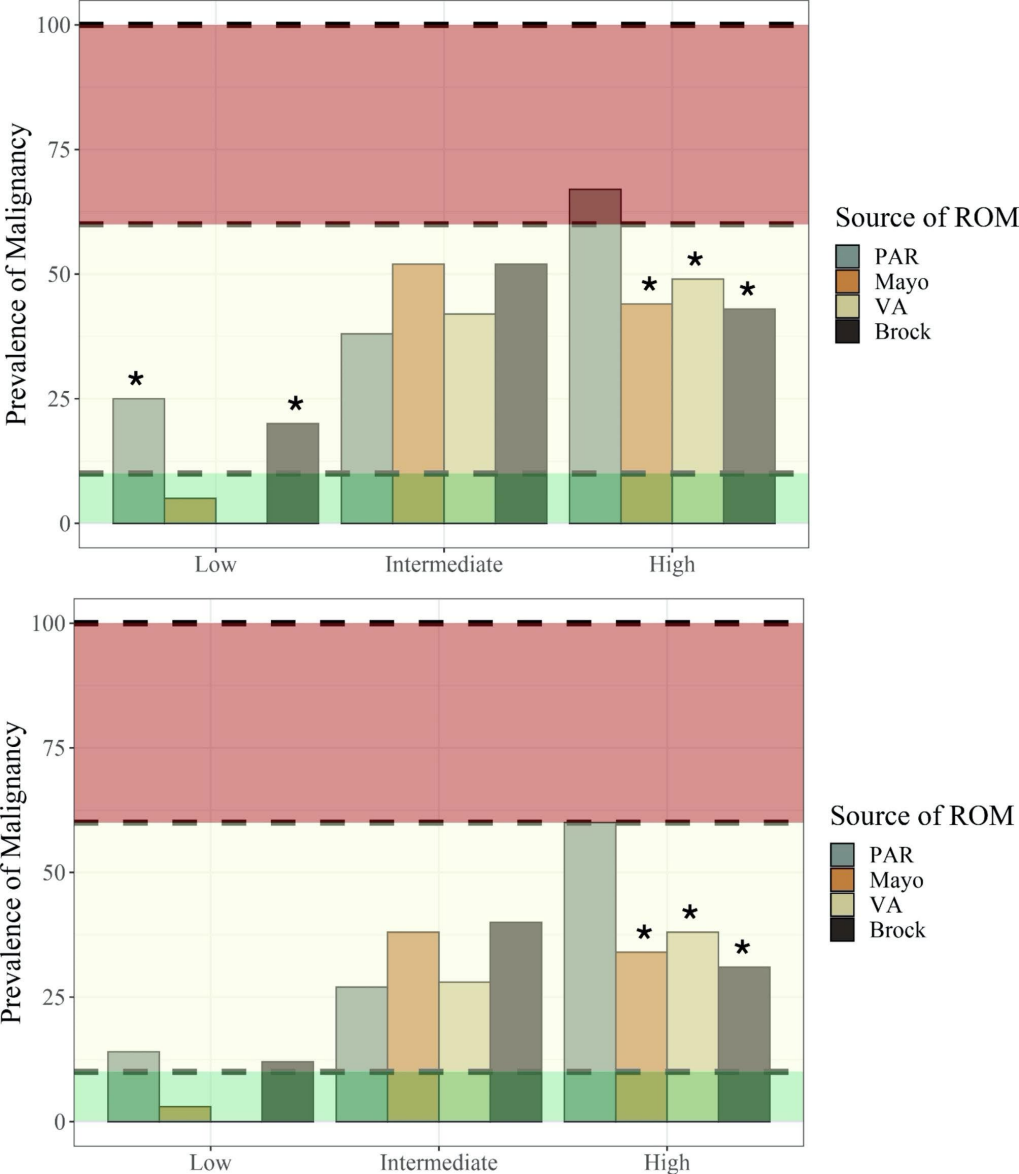
Prevalence of malignancy in groups categorized as low risk (< 10%), intermediate risk (10–60%) and high risk (> 60%) by PAR and three risk models. Data is presented excluding cases that were considered nondiagnostic at 12 months follow up **(**Fig. 1a**)** and with nondiagnostic cases considered to be benign **(**Fig. 1b**)**

Figure 1 illustrates the proportional classification of all 4 modalities to their final adjudicated diagnosis.

PAR = Physician Assessed Risk.

* value is significantly different from the bound of that interval by one-tailed *p-*test.

Due to the high proportion of adjudicated patients that remained nondiagnostic at 12 months interval follow-up, the data was analyzed in two ways. First, nondiagnostic cases were excluded from the analysis (Table 2; Fig. 1a); this analysis likely resulted in an underrepresentation of benign lesions[1].

**Table 2:**
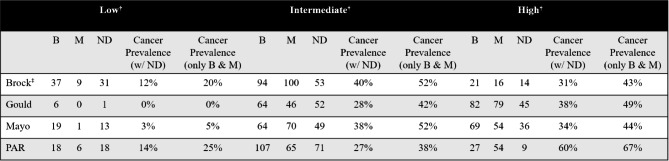
Classification of benign, malignant and nondiagnostic nodules in low, intermediate and high risk groups by PAR and three risk classifiers (total n-375; 125 malignant, 152 benign, 98 nondiagnostic)

† Clinical probability of cancer: Low: < 10%; Intermediate; 10–60%; High: > 60%.

‡ Brock refers to model 2b.

B = benign, M = malignant, ND = nondiagnostic.

**Fig. 2:**
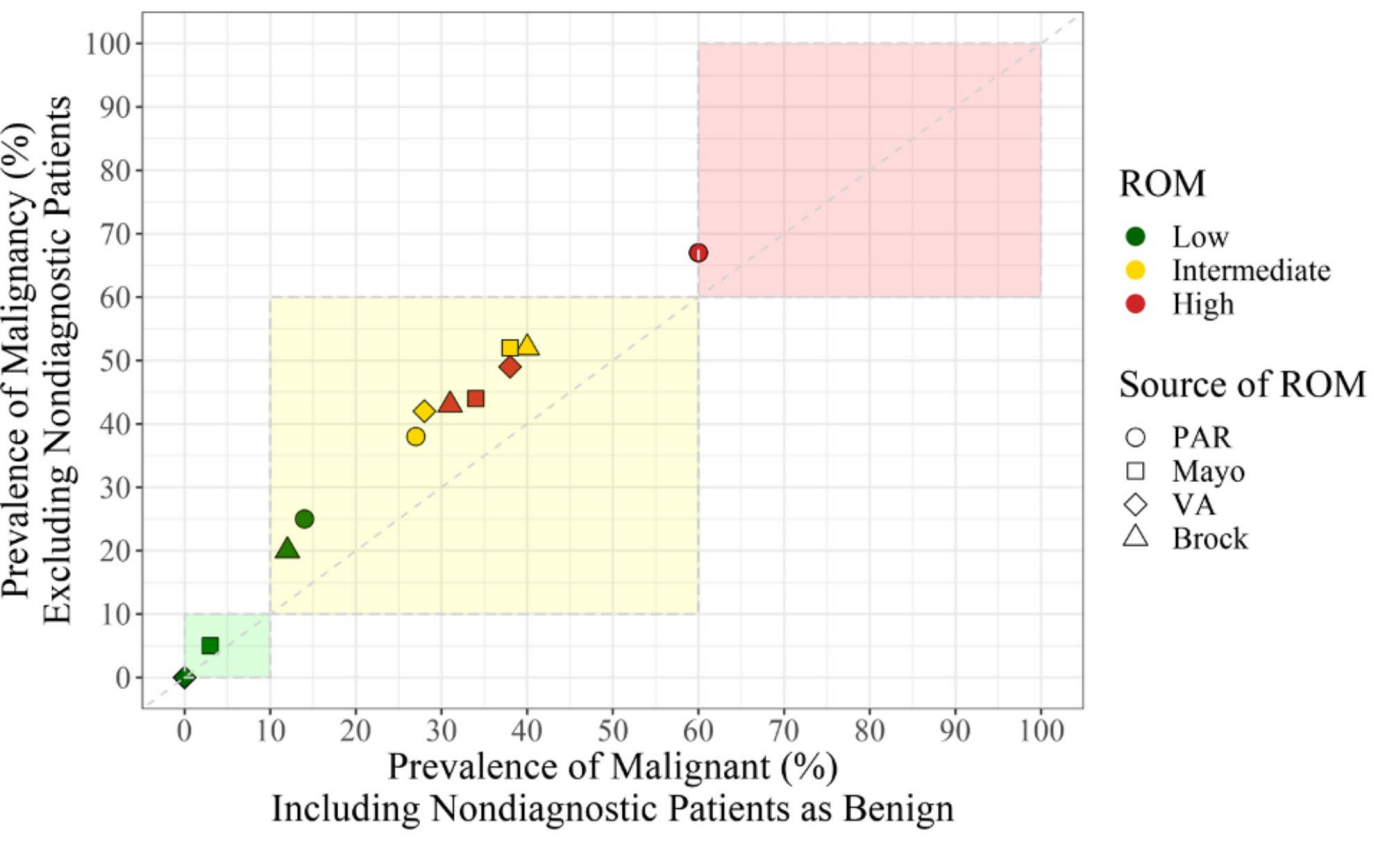
The prevalence in each risk category with the nondiagnostic cases included as benign plotted against the prevalence with them excluded, showing a strong correlation between both methods of analysis

In the second analysis, nondiagnostic cases were assumed to be benign (Table 2; Fig. 1b). As expected, the cancer prevalence in the three risk categories was higher in the analysis where nondiagnostic cases were excluded, given the smaller denominator. To determine whether the status of the nondiagnostic assumed to be benign had a significant effect on the analysis, we plotted the prevalence in each risk category with the nondiagnostic cases included against the prevalence with them excluded (Fig. 2). The result showed a strong correlation between both scenarios. Prevalence data is presented as a range between the two methods of analysis.

ROM = Risk of Malignancy; PAR = Physician Assessed Risk.

**Fig. 3:**
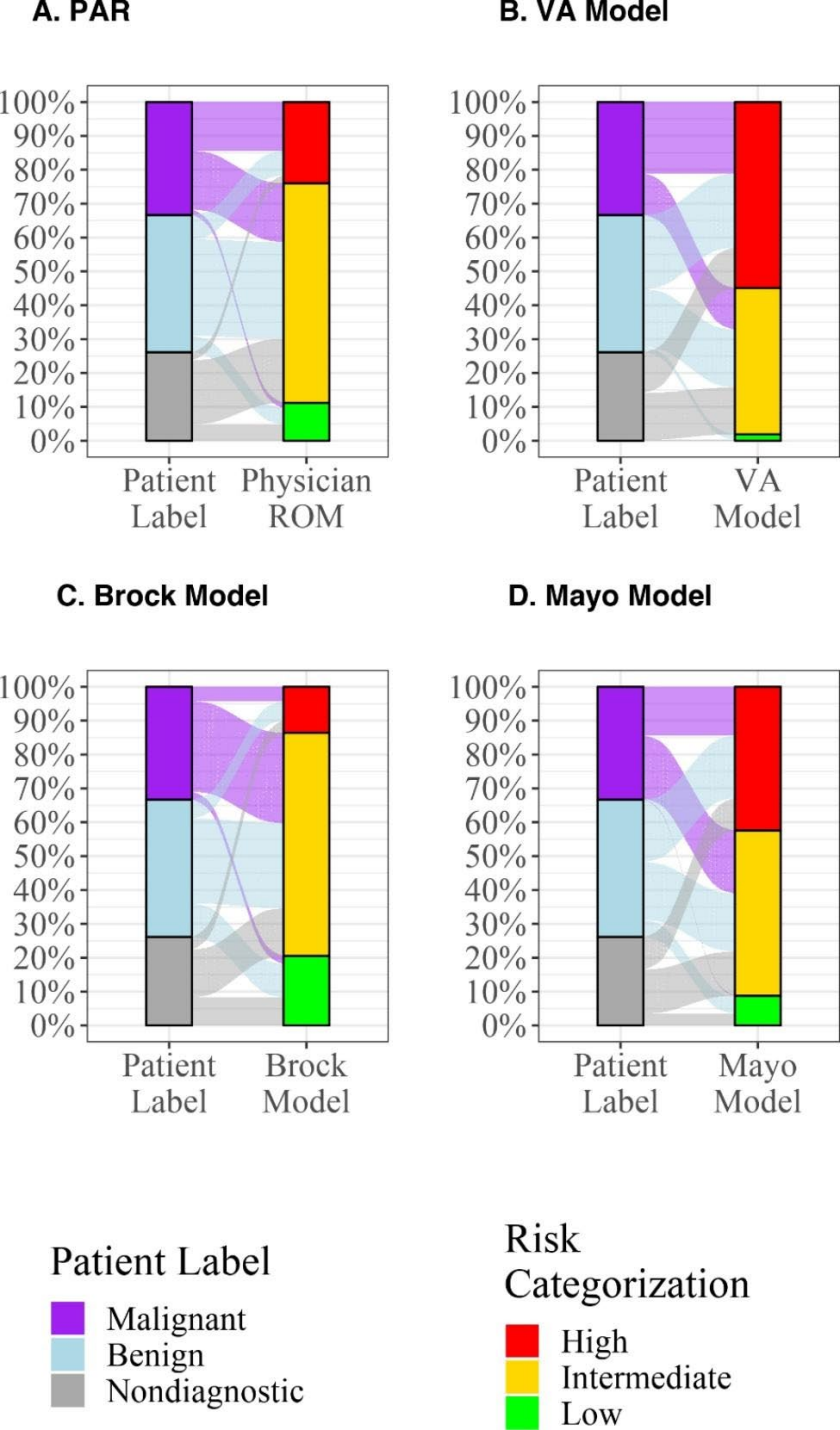
Alluvial Plots showing the risk categorization for Physician Assessed Risk (PAR) and the three risk models, each with their corresponding diagnosis with ≥ 12 months follow up

Figure 3 utilizes alluvial plots to show the distribution of malignant, benign, and nondiagnostic cases in low, intermediate, and high risk categories for each of the risk assessment modalities.

### Patients classified low risk for malignancy: clinical probability of cancer < 10%

The VA Model classified none of the malignant patients as low risk, resulting in a cancer prevalence in this risk category of 0%. However, this model identified only 6 (3.9%) of the 152 truly benign patients as low risk. The Mayo Model classified only 1 (0.8%) of the 125 malignant patients as low risk, with an overall prevalence of malignancy in the Mayo low risk category of 3 − 5%. Both PAR and the Brock Model had a prevalence of malignancy in their low risk category that exceeded the 10% threshold of “low risk”. The Brock Model classified 9 (7.2%) of the malignant patients as low risk, with an overall prevalence of malignancy in its low risk category of 12 − 20%. PAR classified 6 (4.8%) of the malignant patients as low risk, with an overall prevalence of malignancy in its low risk category of 14 − 25%.

### Patients classified intermediate risk for malignancy: clinical probability of cancer 10–60%

All three risk models and PAR had a cancer prevalence in their intermediate risk group that fell within 10–60%. PAR classified 65 (52%) of the 125 malignant patients as intermediate risk, with an overall prevalence of malignancy in intermediate risk category of 27 − 38%. The Brock Model classified 100 (80%) of the malignant patients as intermediate risk, with an overall prevalence of malignancy in intermediate risk category of 40 − 52%. The VA Model classified 46 (37%) of the malignant patients as intermediate risk, with an overall prevalence of malignancy in intermediate risk category of 28 − 42%. The Mayo Model classified 70 (56%) of the malignant patients as intermediate risk, with an overall prevalence of malignancy in intermediate risk category of 38 − 52%.

### Patients classified high risk for malignancy: clinical probability of cancer > 60%

Of the four risk assessment modalities, only PAR had a cancer prevalence in its high risk category that met or exceeded 60%, with a prevalence range of 60 − 67%. All three risk models had prevalence of malignancy in their high risk category of less than 60%, and more than half of all the lesions labeled high risk by the risk models were benign. The Brock Model classified fewer malignant patients as high risk than any other model with a prevalence range of 31 -43%. It correctly labeled only 16 (13%) of the 125 malignant patients as high risk. The VA Model had a prevalence of 38 − 49% in its high risk category, correctly labeling 79 (63%) of the malignant patients as high risk. The Mayo Model had a range of 34 − 44%, categorizing 43% of the malignant patients as high risk. PAR categorized also labeled only 43% of the malignant patients as high risk, yet only 27 (18%) of the benign patients were classified as high risk.

We performed a comparison of PAR to the risk models with respect to the benign and nondiagnostic lesions that were labeled as high risk. The VA model was more likely to misclassify benign or nondiagnostic lesions as high risk in older patients (p = 0.01) and in patients with large lesions (> 3 cm) (p ≤ 0.01) and those with irregular borders (p ≤ 0.01). The Mayo model was more likely to misclassify benign or nondiagnostic lesions as high risk based on nodule characteristics of large size (> 3 cm) (p ≤ 0.01) and irregular borders (p = 0.02). The Brock model also identified many benign or nondiagnostic lesions as high risk based on large size (> 3 cm) (p < 0.01).

**Fig. 4:**
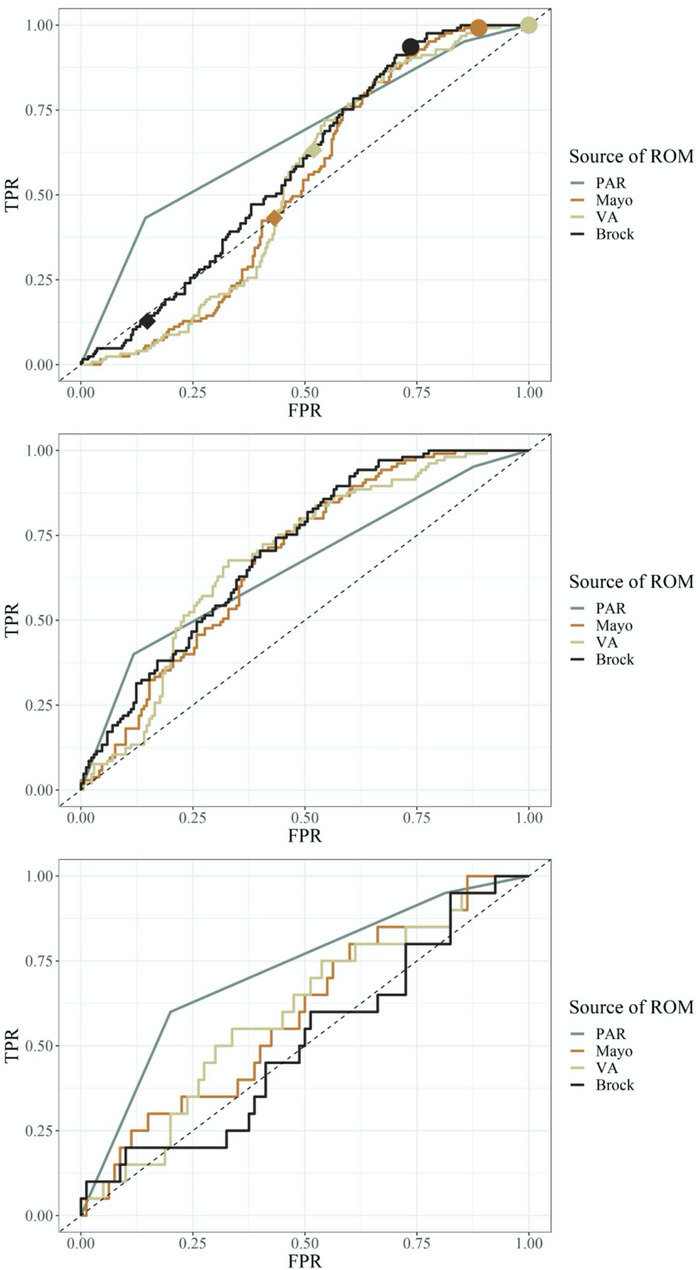
Receiver Operating Characteristic Curve with nondiagnostic patients included as benign **(**Fig. 4a**)**, in patients with lung nodules (lesions ≤ 30 mm) **(**Fig. 4b**)** and in patients with lung masses (lesions > 30 mm) **(**Fig. 4c**)** **a.** (AUCs: Brock = 0.58, VA = 0.53, Mayo = 0.52, PAR = 0.66) **b.** (AUCs: Brock = 0.71, VA = 0.69, Mayo = 0.68, PAR = 0.66) **c.** (AUCs: Brock = 0.51, VA = 0.60, Mayo = 0.59, PAR = 0.72) □ intermediate/high boundaries for each classifier  ○ low/intermediate boundary for each classifier PAR = Physician Assessed Risk.

ROC curves are shown when nondiagnostic cases were considered to be benign for all patients, patients with lung nodules (lesions ≤ 30 mm) and in patients with lung masses (lesions > 30 mm) (Fig. 4).

All three risk models underperformed with respect to the AUC compared to their original published results when applied to this population. The Brock, VA and Mayo models had AUCs of 0.58, 0.53 and 0.52, respectively. PAR only slightly outperformed the three models with an AUC of 0.66.

### Performance in lung nodules vs. lung masses

When the analysis was confined to lung nodules (lesions ≤ 30 mm), each of the risk calculators showed slightly improved AUCs compared to their performance in the larger cohort, but these remained below the AUCs reported in their respective validation cohorts (Fig. 4b and c). When lung masses were considered separately, PAR outperformed the risk calculators, with an AUC of 0.72 compared to 0.51, 0.60, and 0.59 for Brock, VA, and Mayo, respectively.

### Performance of the models using their intended risk thresholds for low, intermediate, and high risk

**Fig. 5:**
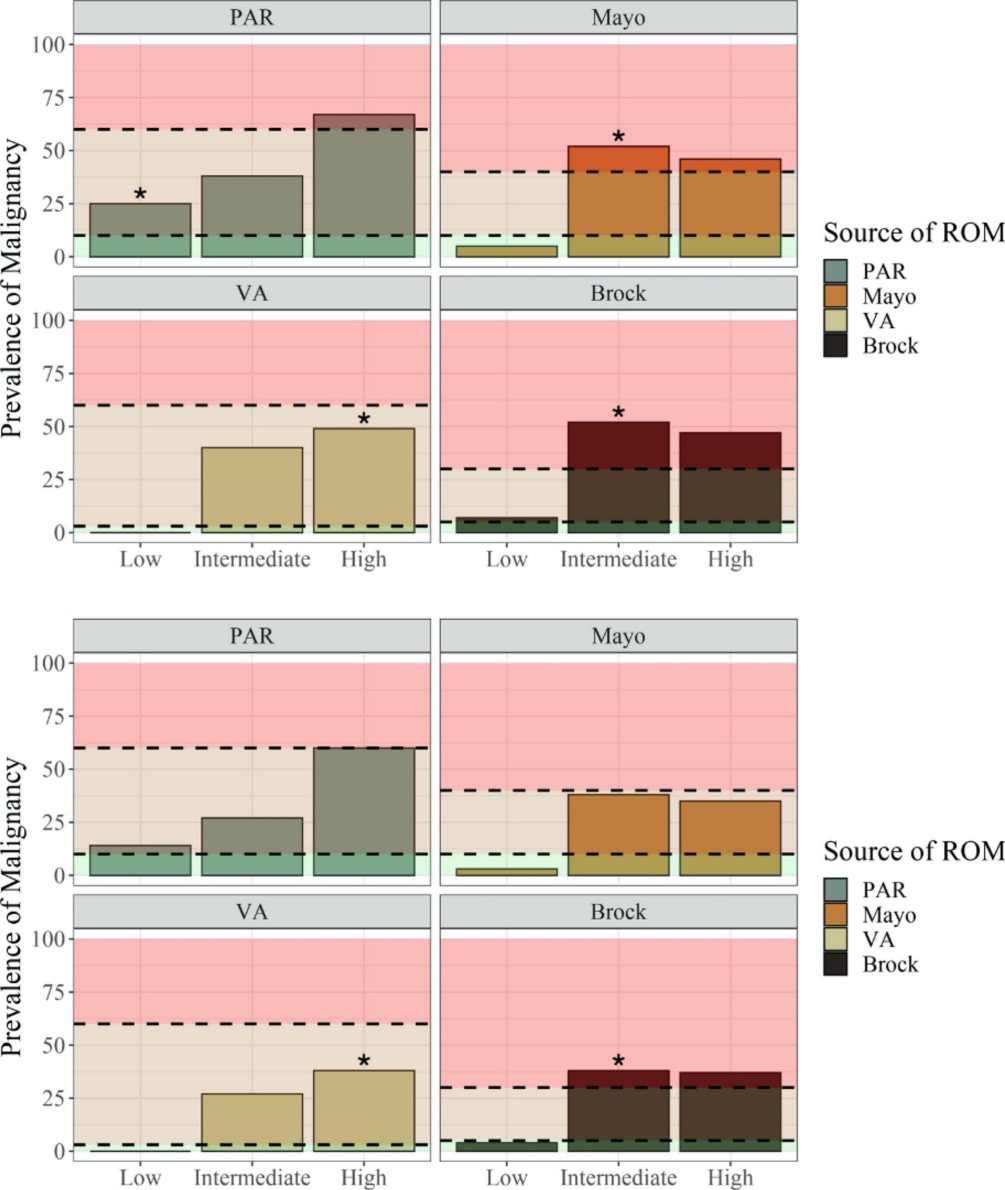
Prevalence of malignancy in groups categorized as low risk, intermediate risk, and high risk by PAR and three risk models utilizing each model’s original thresholds. Data is presented excluding cases that were considered nondiagnostic at 12 months follow up **(**Fig. 5a**)** and with nondiagnostic cases considered to be benign **(**Fig. 5b**)**

Each of the risk models utilized different thresholds for what was considered to be low, intermediate, and high risk in their original validation cohorts. We analyzed our data using each model’s intended risk thresholds for risk categorization (Fig. 5). The Mayo model regarded low risk as < 10% and high risk as > 40%[16]. At those thresholds, the Mayo model had a significantly higher prevalence of cancer in the patients labeled intermediate risk when nondiagnostic patients were excluded. The VA model utilized < 3% and > 60% for low and high risk, respectively[17]. No patients with cancer were labeled low risk by this model at this threshold. The prevalence of malignancy in the group of patients labeled high risk by the VA model was significantly lower than expected irrespective of whether nondiagnostic cases were included or excluded. Finally, the Brock model was analyzed at thresholds of < 5% and > 30% for low and high risk, respectively. The prevalence of malignancy in the group of patients labeled intermediate risk by the Brock model was significantly higher than expected irrespective of whether nondiagnostic cases were included or excluded.

PAR = Physician Assessed Risk.

* value is significantly different from the bound of that interval by one-tailed *p-*test.

## Discussion

Our data shows that PAR and all three clinical risk model calculators do a poor job overall of appropriately categorizing the risk of malignancy in patients undergoing bronchoscopy for suspected lung cancer where the pathology is nondiagnostic. Although the risk models matched the expected prevalence in the intermediate risk group, more than half of all the lesions labeled high risk by all three risk models were truly benign. Use of these models in this subset of patients to guide decision-making could result in patients with benign disease undergoing repeat bronchoscopy, other invasive non-surgical sampling, or surgery.

The accuracy of a risk model calculator in an individual patient is influenced by the degree to which that patient matches the characteristics of the population used to develop the model. Risk models that directly incorporate the root causes of the disease, such as genomic alterations, are often more generalizable and robust to small cohort changes. On the other hand, models built solely on correlating factors that only have an indirect role in the disease are often more sensitive to changes when the composition of those factors changes in the patient cohort. Our data suggest that the subgroup of patients with lung lesions who undergo bronchoscopy may not have been well-represented in the training and validation sets for these models, limiting their usefulness in clinical practice.

Multiple studies have sought to compare PAR and various risk models. One recent prospective study concluded that clinician assessment was slightly better at predicting malignancy than two validated models[8], while other studies show similar accuracy between PAR and various models[6, 18]. In one survey study that used clinical vignettes to test the accuracy of risk assessment, all modalities showed only modest performance, with PAR showing an AUC of only 0.70 (95% CI, 0.62–0.77), while the performance of two commonly used risk models was essentially no better[18].

PAR in this study demonstrates a pattern of categorization that better matches the expected prevalence of malignancy in each risk category. However, it too performed suboptimally in this group of patients. While PAR slightly outperformed the risk models with respect to the ROC curves, PAR’s AUC of 0.64 (excluding nondiagnostic lesions) demonstrates relatively poor discriminatory performance, whereas the AUCs for the Brock, VA and Mayo Models sit at, or close to, random chance for the study population, and only slightly better when confined to lung nodules. None of the risk calculators came near to approximating the AUCs demonstrated in their respective validation sets, lacking specificity in this cohort of patients, evidenced by the S-shaped ROC curves for the Mayo and VA model. In the lower left quadrant of an ROC, where the curve reflects specificity, the plots for Mayo and VA fall to the right of the line where the true positive rate and true negative rate are equal. Thus, these risk model calculators are wrong more often than they are right when labeling a lesion high risk in this population of patients. The Brock model performs only as well as random chance. Strongly driven by size, these models are likely to interpret smaller cancers as benign while categorizing larger benign lesions as likely malignant. The larger, spiculated lesions in older patients that ultimately proved not to have cancer likely represented inflammatory or infectious findings on CT. The slightly superior performance of PAR in this subset of patients may reflect the ability of a physician to weigh clinical context as part of the risk assessment. The models perform better with respect to their categorization of patients as high-risk when utilized with their intended thresholds used in their validation, as might be expected. These thresholds are not generally used in clinical practice, nor do the current ACCP guidelines recommend using an individual model’s original thresholds to guide decision making[4].

Inaccurate risk assessment contributes significantly to health care costs. In a recent cost benefit analysis using CMS claims data, 43.6% of patients who underwent a biopsy for a suspicious lung lesion were found not to have a cancer. Over 43% of the total costs of diagnostic evaluations for lung cancer in the U.S. was attributable to invasive procedures performed in patients with benign disease[19]. With the growing acceptance of lung cancer screening and increased use of diagnostic CT for other reasons, we can expect to see greater numbers of patients with lung lesions that will require risk assessment[1, 20]. Understanding the accuracy and limitations of the currently available tools for risk assessment in these patients will be crucial for the appropriate utilization of resources to meet the needs of this epidemic, facilitating prompt diagnosis and treatment for patients with cancer while minimizing morbidity and cost for those without.

Our study is the first to evaluate the performance of PAR and the risk model calculators in this subset of patients deemed appropriate for bronchoscopic biopsy where the effort has failed to establish a diagnosis. It has several important strengths. First, it demonstrates the scope of the problem in real-world clinical practice: 60% of cases in the Percepta BGC Registry Study had a nondiagnostic bronchoscopy. It focuses on this subset of group of patients in whom decision-making regarding management of suspicious lung lesions is often most difficult – those where non-surgical tissue sampling is deemed necessary but has failed once, necessitating further invasive procedures or a retreat to radiographic surveillance. Another strength is the fact that PAR was determined and recorded by the treating pulmonologist prior to the procedure, thus our PAR reflects real world clinical practice.

Our study has several limitations that are important to consider when attempting to generalize the findings to other patients undergoing risk assessment for lung cancer. Each of the models was developed and validated on a specific population, and none were developed specifically in a cohort of patients undergoing bronchoscopy, thus their suboptimal performance in our cohort may be expected. Moreover, the two-conditional nature of our cohort, with patients first selected for bronchoscopy and then selected for a nondiagnostic result, may have resulted in a prevalence of malignancy in the high risk category that was lower than the prevalence in the group of patients undergoing bronchoscopy as a whole. If, for example, a risk model was very good at correctly identifying larger malignant lesions as high risk, and bronchoscopy was more likely to be successful in these patients, the result would be a reduction in the prevalence of malignancy in that model’s high risk category in the nondiagnostic subset. This kind of bias could account for the differential performance of the models in lung nodules compared to lung masses. The fact that our findings may be an underestimate of the performance of the models in lesions undergoing bronchoscopy as a whole does not diminish their importance; a nondiagnostic bronchoscopy is a common, real-world scenario in which decision-making is guided by risk assessment, and accuracy in this assessment is essential.

The finding that patients with cancer were less likely to have large (> 30 mm) lesions is somewhat surprising, given that ROM increases with increasing size, with 30 mm representing an accepted threshold for a high ROM[4]. A form of inclusion bias may account for this particular observation in this study. Larger, malignant lesions are more likely to be associated with nodal metastases than smaller malignant lesions. In standard practice, patients with both a mass and suspicious lymphadenopathy are likely to have their diagnosis (and stage) established using endobronchial ultrasound guided fine needle aspiration rather than a forceps biopsy of the mass. These patients, therefore, are unlikely to have been enrolled in a study of patients undergoing standard bronchoscopy for a peripheral lung lesion. This could bias the subset of larger lesions in our study toward benign infection or inflammatory lesions. Another limitation is that we did not capture any longitudinal imaging data in this study. The data captured for this study included only the lesion size at the time of the bronchoscopy, not size change over time. Each of the patients included here represented a patient in whom the pulmonologist thought that the suspicion for malignancy was high enough to justify bronchoscopic sampling given the clinical and radiologic context, but the impact of any serial imaging on a determination of ROM is not discernible in our data.

Another important consideration is the fact that the Mayo and VA risk models were developed in patients with pulmonary nodules defined as ≤ 30 mm; patients with lesions > 30 mm were not included in their validation cohorts. 26% of our study cohort had lesions > 30 mm and therefore fell outside of the confines of these models. Weakness of these models in patients who fall outside the confines of their validation cohorts might be expected, but the Brock model was developed in a cohort with lesions up to 86 mm, and it too performed poorly in our study. It is worth noting that the online calculators based on these models accept input for size exceeding 30 mm. Also worth noting is the fact that the Brock model was developed in a cohort of screening patients where the cancer prevalence was considerably lower than the prevalence in our study, which would alter the performance of the model in our study cohort. While it is possible to recalibrate the model for a higher prevalence, we chose to use the published model, as would be done in an individual patient in real-world risk assessment.

Another limitation of our study is the fact that the cut points used in this study do not conform to the cut points used in the current ACCP guidelines for lung nodule management[4]. This was a limitation imposed by our source data, which was collected as part of a larger trial that collected only categorical data on physician assessed risk (PAR), using 10% and 60% for low and high risk categories. The intent of this study was not to say what should or should not have happened given a certain level of pre-test risk; the main intent was to show that PAR and the risk calculators under- or over-estimate risk in certain risk categories.

What is needed is an objective, less error-prone means for risk assessment that can be used across the heterogeneous spectrum of patients with indeterminant lung lesions. Optimally, this would be an accurate, noninvasive biomarker that could further enhance the utility of clinical and radiographic factors in differentiating early-stage lung cancers from benign disease[7, 21]. There have been remarkable advances in lung cancer biomarkers that variably employ blood-based testing, genomic testing, or artificial intelligence to improve on the performance of the clinical-only risk models. Various novel methods for risk assessment have been developed that utilize plasma biomarkers, autoantibodies to tumor-associated antigens, exhaled breath compounds, and bronchial and nasopharyngeal genomic classifiers, to augment the accuracy of existing risk prediction models in identifying malignant lesions from benign disease[22–26]. Radiomics, the use of quantitative data obtained from CT imaging to predict the risk of malignancy in a nodule, is another approach actively in development[27]. Each of these novel tools will require both clinical validation and a determination of clinical utility before they can be incorporated into the paradigm for risk assessment of patients with indeterminant lung lesions, with the potential to improve care for both patients with lung cancer and those without[7, 28].

## Conclusion

Though multiple validated risk prediction models exist to guide the management of patients with an indeterminant pulmonary lesion, the accuracy of these models and physician-assessed risk is suboptimal in patients in whom bronchoscopy is being considered given the potential for a nondiagnostic result. While PAR outperformed the risk models overall, it also lacked the level of accuracy that would be desirable for optimal risk stratification to guide decision-making. Better tools are needed for assessment of the risk of malignancy in patients with pulmonary lesions.

## Data Availability

The datasets generated during the current study are not publicly available due to concerns regarding participant confidentiality and proprietary information but are available upon reasonable request from the corresponding author.

## Abbreviations

PAR: physician-assessed risk.
ROM: risk of malignancy.
BGC: Bronchial Genomic Classifier.
VA: Veterans Affairs.
AUC: area under the curve.
